# Evaluation of cardioprotective effect of silk cocoon (Abresham) on isoprenaline-induced myocardial infarction in rats

**Published:** 2013

**Authors:** Ritesh Kumar Srivastav, Hefazat Hussain Siddiqui, Tarique Mahmood, Farogh Ahsan

**Affiliations:** 1 *Department of Pharmacology, Faculty of Pharmacy Integral University, Lucknow (U.P), India*; 2 *Advisor to V.C. Integral University, Lucknow (U.P), India*

**Keywords:** Abresham, *Bombyx mori*, Cardioprotective Effect, Isoprenaline

## Abstract

**Objective**: The study was conducted to evaluate cardioprotective effect of silk cocoon (Abresham) *Bombyx mori *(*B. mori*) on isoprenaline-induced myocardial infarction. This study deals with the cocoons, which is called Abresham in the Unani system of medicine. It is one of the 64 drugs which Avicenna has mentioned in Avicenna’s tract on cardiac drugs and used in the treatment of cardiovascular diseases. Abresham is a chief ingredient of the two very famous Unani formulation viz. Khamira Abresham Sada, and Khamira Abresham Hakim Arshad Wala.

**Materials and Methods: **The ethanolic extract of *B. mori* (Abresham) silk cocoons in the dose of 250 mg/kg and 500 mg/kg body weight was administered orally for 28 days before isoprenaline administration to test their cardioprotective effect. Isoprenaline (85 mg/kg) was administered subcutaneously on days 29^th^ and 30^th^, respectively in order to induce myocardial infarction.

**Results:** The parameters for evaluation of cardioprotective activity were the physical parameters and the biochemical estimations. The physical parameters were gross examination of heart, heart weight/body weight ratio and histopathology examination. In biochemical estimations, the activity of various cardiac enzymes such as aspartate transaminase, alanine transaminase, creatinine kinase, lactate dehydrogenase, and the gold marker troponin-I were determined. The levels altered by isoproterenol were restored significantly by the administration of the both doses of test extract especially at higher dose.

**Conclusion: **The result of this study shows that alcoholic extract *B. mori* has significant cardioprotective activity against isoprenaline-induced myocardial infarction.

## Introduction

Myocardial infarction (MI) is a major clinical problem and a leading cause of death throughout the world. Developing countries such as India are struggling to manage the impact of infectious diseases simultaneously with growing burden on society and health system caused by non-communicable diseases such as myocardial infarction. An increasing number of young Indians are succumbing to myocardial infarction (Suchalata and Shyamala, 2004 ▶). Myocardial infarction is the rapid development of myocardial necrosis caused by critical imbalance between the oxygen supply and the demand of myocardium. Oxidative stress resulting from increased production of free radicals associated with decreased levels of antioxidants in the myocardium plays a major role in cardiovascular diseases. isoprenaline (ISO), a catecholamine, was administered in the present study, as it serves as a standard model to study the beneficial effect of many drugs on cardiac function. ISO is a synthetic β-adrenergic agonist that causes severe stress in the myocardium and causes necrosis in the heart muscle. ISO-induced myocardial necrosis showed membrane permeability alterations, which bring about the loss of function and integrity of myocardial membranes (Siddiqui, 1962 ▶).

*B mori* is a worm which feeds on the leaves of Morus. The cocoons are oval sacs and their covering is spun by a group of silk moths during their metamorphosis. Internally the sac contains dark-brown dried remains of a caterpillar. The study deals with the cocoons, which is called Abresham in the Unani system of medicine. In this system, Abresham is a drug of great repute in treating respiratory and cardio-vascular disease and is the chief ingredient of the two very famous Unani formulation viz. Khamira Abresham Sada, Khamira Abresham Hakim Arshad Wala. All the published work has been done on the silkworm, which is reported to contain amino acids, nucleoproteins, and nucleic acids, but so far, no more pharmacological works has been carried out on the cocoons. (Subhasini, 2011 ▶)

## Materials and Methods

**Plant material**

The Silk cocoons, *B. mori *were purchased from the local market of Lucknow. The drug was authenticated by Division of Taxonomy, National Botanical Research Institute Lucknow (UP) (Ref No. NBRI/CIF/270/2011 and Specification NBRI-SOP-202).

**Preparation of extract**

The clean fresh cocoon shells of the silk worm *B. mori* were cut into small pieces convenient for the purpose of extraction. The dark brown remains of the caterpillar were removed from the cocoons. The drug was extracted with 80% ethanol. When extract was concentrated by evaporation to half of the volume, a waxy material separate out and was filtered. The filtrate on further concentration to half of the volume gave a dark brown resinous residue. The concentrated extract was weighted according to the body weight, and accurately dissolved in normal saline for dose treatment daily (Subhasini N., 2011 ▶).

**Animals**

Albino Wistar rats of 125-150 g of either sex were used for the study. The inbred species of rats were obtained from animal house of Central Drug Research Institute (CDRI), Lucknow for experimental purpose. The animals were maintained under controlled conditions of temperature (23±2 ºC) before the study. The animals were randomized into experimental, normal, and control groups, housed individually in sanitized polypropylene cages containing sterile paddy husk as bedding. They had free access to standard pellets as basal diet and water *ad libitum*. Animals were habituated to laboratory conditions for 48 hours prior to experimental protocol to minimize any non-specific stress. All the studies conducted were approved by the Institutional Animal Ethical Committee (IAEC) of Faculty of Pharmacy, Integral University, Lucknow (Registration No. IU/Pharm/M.pharm/CPCSEA/12/12), India. 

**Chemicals**

Normal saline (0.9%) (Albert David Ltd, Ghaziabad), Isoprenaline Hydrochloride, Metoprolol Tartrate (Pure drug) (Sigma Chemicals, USA), Petroleum Ether (40-60) (S.D.Fine Chemicals, Mumbai), Ethanol (Jiangsu Huaxi Ltd, China), Formaldehyde (Fisher scientific Ltd, Navi Mumbai), Serum ALAT diagnostic kit, Serum ASAT diagnostic kit, Serum Creatinine diagnostic kit (Span Diagnostics Ltd, Surat), Serum LDH diagnostic kit (Accurex Biomedical Pvt Ltd Thane, Mumbai), and all the chemicals used were of analytical grade.

**Experimental Procedure**

Group I– Termed as normal control group (NC group), received distilled water (0.5 ml, orally) daily for 30 days.

Group II– Termed as isoprenaline group (ISO group), received distilled water (0.5 ml, orally) daily for 30 days, in addition, received injections of ISO (85 mg/kg, s.c.) at an interval of 24 hour on 29^th^ and 30^th^ day. Group III– Termed as standard group (STD group), received metoprolol (pure) (10 mg/kg/day, orally) daily for 30 days and in addition received ISO (85 mg/kg, s.c.) on the 29^th^ and 30^th^ days at an interval of 24 hours. Group IV–Termed as test group 1 (TG1), *B. mori* extract (250 mg/kg/day, orally) administered daily for 30 days and in addition received ISO (85 mg/kg, s.c.) on the 29^th^ and 30^th^ days at an interval of 24 hours. Group V – Termed as test group 2 (TG2), *B. mori* extract (500 mg/kg/day, orally) administered daily for 30 days and in addition received ISO (85 mg/kg, s.c.) on the 29^th^ and 30^th^ days at an interval of 24 hours.

ISO = Isoproterenol

*Each group contained 5 animals (either sex) 

**Induction of myocardial infarction**

Myocardial infarction was induced by 85 mg/kg body weight of isoprenaline hydrochloride, dissolved in normal saline and given through subcutaneous injection for two consecutive days (29^th^ and 30^th^) (Murugesan et al., 2009 ▶). 

Rats were weighed and put down 24 hours after the final subcutaneous injection of ISO. Blood collection was done by adhering to Good Laboratory Practices. Blood was collected through retro-orbital plexus from the inner canthus of the eye using capillary tubes and cardiac puncture under light ether anesthesia and allowed to clot for 30 minutes at room temperature. The serum was separated by centrifugation at 3000 rpm at 30 °C for 15 minutes and used for the estimation of marker enzymes, including aspartate aminotransferase (AST), lactate dehydrogenase (LDH), and creatine phosphokinase (CPK). All animal were sacrificed by cervical decapitation. The hearts were dissected out immediately, weighed, and then fixed in 10% buffered neutral formalin solution (Rona G. Et al., 1959 ▶). 

**Measured parameters**

*Gross examination of rat heart*

The dissected hearts were washed with ice cold saline. They were visually examined for inflammation, redness, scar formation, and colour in all parts and grading was performed (Sarvanan et al., 2010 ▶) as follows. 

Grade 0= No Lesion

Grade 1= Inflammation, redness, and capillary dilations.

Grade 2= Edema and yellowish ventricle portion

Grade 3= Atrium and ventricle turns yellow and scar formation

Grade 4= Diffuse heart, absolute scar formation, and increased necrosis portion

**Heart weight/body weight ratio**

Each rat was euthanized, weighed, and total body weight recorded. The rat was placed on its back and pinned onto board with extended extremities (inner side of hands and feet). The rat was wiped or wetted with 70% ethanol to control hair and dander. Removal of the heart was performed by dissecting the aortic root immediately above the aortic valves and the superior vena cava above the atria. Heart blood was removed from heart and washed with ice cold saline. The dry heart was weighed and recorded. Then, the heart was place in fixative (Firoz et al., 2011 ▶). 

**Biochemical estimations**

At the end of the experimental period, the blood samples were taken and serum was separated for analysis of different enzymes related to myocardial infarction: lactate dehydrogenase (LDH), creatine kinase-MB fraction (CK-MB), aspartate transaminase (AST), and alanine transaminase (ALT). All the analyses were performed with commercially available kits purchased from Span Diagnostics Ltd, Surat, Accurex Biomedical Pvt Ltd Thane, Mumbai and measured spectrophoto-metrically, Shimadzu. Release of troponin-I was estimated by troponin –I Rapid test kit commercially purchased from Reckon Diagnostic Pvt Ltd (Rona et al., 1959 ▶). 

**Histopathological study (Microscopic)**

At the end of the study, the heart was isolated and washed with ice cold saline. The tissue was fixed in 10% buffered neutral formalin solution. After fixation, tissues were embedded in paraffin-wax and five micrometer thick sections were cut and stained with hematoxylin and eosin. The slides were observed under light microscope and photomicrograph was taken (The microscopy was done by Capital pathology, Lucknow).

**Statistical analysis**

Statistical analysis was performed using one way ANOVA followed by Dunnett’s t test (GraphPad software Instat, USA).

## Results

**Gross examination of heart**

The visual examination of the heart seeking for inflammation, redness, capillary dilatation, scar formation, and colour change was performed and grading of the heart was done. The isoprenaline (ISO) group showed marked inflammation, scar formation, and diffused heart when compared with the normal control (NC) group. The standard (STD) group showed reduction in edema, capillary dilation, and scar formation, with little redness when compared with the isoprenaline (ISO) group. The test group 2 (TG2) (500 mg/kg) showed remarkable decrease in inflammation, redness, capillary dilatation, and scar formation as compared with the test group 1 (TG1) (250 mg/kg) when both extracts were compared with the isoprenaline (ISO) group (Table 1).

**Table 1:** Observation table for grading of heart

**Groups**	**Grading of cardiac damage**
**Normal control (NC)**	Grade 0
**Isoprenaline (ISO)**	Grade 4
**Standard (STD)**	Grade 1
**Test group 1 (TG1) (250mg/kg)**	Grade 3
**Test group 2 (TG2) (500mg/kg)**	Grade 2

**Heart weight/ body weight ratio**

The heart weight and heart weight/body weight ratio was analyzed in various treatment groups. The isoprenaline (ISO) group showed marked increase in heart weight due to hypertrophy, when compared with the normal control (NC) group. The standard (STD) group showed reduction in heart weight and heart/body weight ratio when compared with the isoprenaline (ISO) group. The test group 2 (TG2) (500 mg/kg) demonstrated decrease in heart weight and decrease in heart/body weight ratio when compared with the isoprenaline group. When both test groups (250 mg/kg, 500 mg/kg) were compared with the isoprenaline (ISO) group, the highest dose (TG2) (500 mg/kg) showed reduction in heart weight and heart/body weight ratio as compared with the lowest dose (TG1) (250 mg/kg) (Table 2).

**Table 2:** Body weight, heart weight, heart/body weight ratio

**Groups**	**Body weight (g)**	**Heart weight (g)**	**Heart/body weight ratio** **(x 10** ^3)^
**Normal control (NC)**	208	0.81	3.8
**Isoprenaline (ISO)**	191	1.08	5.6
**Standard (STD)**	216	0.88	4.0
**Test group 1 (TG1) (250 mg/kg)**	220	1.01	4.5
**Test group 2 (TG2) (500 mg/kg)**	225	0.98	4.3

**Biochemical estimations**

The effects of oral administration of ethanolic extract of *B. mori* on serum marker enzymes AST, ALT, LDH, and CPK after 30 days are outlined in (Table 3). Rats treated with ISO showed a highly significant increase (p<0.001) in activities of serum marker enzymes compared with the normal rat (NC) group. Rats pretreated with metoprolol (STD group) when compared with the isoprenaline (ISO) group showed a highly significant (p<0.001) reduction in cardiac marker enzyme. Pretreatment with high dose of *B. mori* (TG2) (500 mg/kg) to rats for 30 days, followed by ISO subcutaneous injection on the 29^th^ and 30^th^ days, licited a highly significant (p<0.001) reduction in the ISO-induced increased activities of AST, ALT, LDH, and CPK. The low dose of coleus forskohlii (TG1) (250 mg/kg) when compared with the isoprenaline (ISO) group was significant (p<0.01) in lowering ISO-elevated serum enzyme activities (Table 3). The release of troponin-I was estimated by Rapid test kit after 4 hours of infarction. When comparing the isoprenaline (ISO) group with the normal control (NC) group, all animals of ISO group showed presence of troponin in serum. When the standard group was compared with the isoprenaline group (ISO), more than half of the animals showed absence of troponin in their serum while in the rest of them troponin was found to be present. When both test groups (TG1, TG2) (250 mg/kg, 500 mg/kg) were compared with the isoprenaline (ISO) group, the highest dose (TG2) (500 mg/kg) showed remarkable decreases in release of troponin as compared with the lowest dose (TG1) (250 mg/kg) (Table 4).

**Table 3:** Effect of ethanolic extract of *B.* *mori* on CK–MB, LDH, AST, and ALT levels in rats by isoprenaline-induced cardiac toxicity

**Treatment**	**AST (IU/L)**	**ALT (IU/L)**	**LDH (IU/L)**	**CK (mg/dl)**
**NC**	155.137±24.5	103.630±2.41	107.780±2.11	1.034±0.020
**ISO**	285.911±24.9^a^	255.144±16.9^a^	307.743±22.44^a^	1.260±0.044^a^
**STD**	161.277±23.4^b^	105.226±22.8^b^	111.106±4.27^b^	1.068±0.029^b^
**TG1**	199.65± 18.10^c^	214.25± 3.63^c^	247.72± 7.22^c^	1.26± 0.04^c^
**TG2**	171.85±21.63^d^	140.60± 16.50^d^	238.96± 16.33^d^	1.15± 0.03^d^

Values are mean±SEM for five animals in each group.

AST: Aspartate amino transferaces; ALT: Alanine transaminase; LDH: Lactate dehydrogenase CK-MB: Creatine kinase; NC: Normal control; ISO: isoprenaline; STD: Standard; TG1: Test group 1; TG2: Test group 2.

a p<0.001 When ISO group is compared with NC group;

b p<0.001 When standard group is compared with ISO group;

c p<0.01 When experimental group is compared with ISO group;

d p<0.001 When experimental group is compared with ISO group

**Table 4:** Release of troponin–I in various treatment groups

**Animal no.**	**Normal Control**	**Isoprenaline** **(85 mg/kg)**	**Standard** **(10 mg/kg)**	**Test extract 250 mg/kg**	**Test extract 500 mg/kg**
**1**	-ve	+ve	-ve	+ve	-ve
**2**	-ve	+ve	-ve	+ve	-ve
**3**	-ve	+ve	+ve	-ve	+ve
**4**	-ve	+ve	-ve	+ve	-ve
**5**	-ve	+ve	+ve	-ve	+ve

+ve: Presence of troponin in serum; -ve: Absence of troponin in serum.

**Histopathological examination**

The myocardial tissue was immediately fixed in 10% buffered neutral formalin solution. After fixation, tissues were embedded in paraffin and serial sections were cut and each section was stained with hematoxylin and eosin. The slides were examined under light microscope and microphotographs were taken.

**Figure 1:**
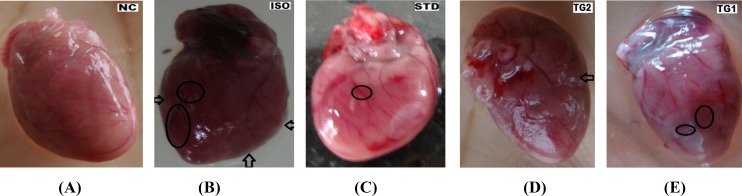
Visual photographs of dissected rat heart: a- (NC) group showing no scar, oedema, or capillary dilation; b- (ISO) group showing hypertrophy, oedema, and change in colour (circle and arrow); c- (STD) group showing no oedema, capillary dilation but showing inflammation and redness (circle); d- (TG1) group showing redness, inflammation near ventricle (circle); e- (TG2) group showing no redness, but shows minor inflammation (arrow).

Photomicrograph of rat heart of the normal control group shows the endocardium, myocardium, and epicardium as well as papillary muscles and vasculature which were all normal and healthy in normal control group. There was no muscular hypertrophy or evidences of myositis (necrosis and/or round cell infiltrates), clearly visible in 10× (prominently) (Figure1 a). Isoprenaline-treated (ISO) group shows focal myonecrosis with myophagocytosis and lymphocytic infiltration. In subendocardium, vacuolar changes and prominent oedema along with chronic inflammatory cells are clearly visible in 10× (prominently) (Figure 1 b).

Rat heart pretreated with the standard (STD) metoprolol (10 mg/kg) shows lesser degree of myonecrosis, myophagocytosis and lymphocytic infiltration, oedema, and very little infiltration of inflammatory cells are clearly visible in 10× (prominently) (Figure 1 c). 

Photomicrograph of rat heart pretreated with *Bombyx mori *(TG1) (250 mg/kg) group shows reduced degree of myonecrosis and lesser infiltration of inflammatory cells but myophagophytosis and subendocardium vacuolar changes are present and clearly visible in 10× (prominently) (Figure 1 d). Pretreated rat heart with *Bombyx mori *(TG2) (500 mg/kg) treated group shows little degree of myonecrosis and lesser infiltration of inflammatory cells as well as a decreased myophagophytosis and subendocardium vacuolar changes are present and clearly visible in 10× (prominently) (Figure 1 e).

**Figure 2:**
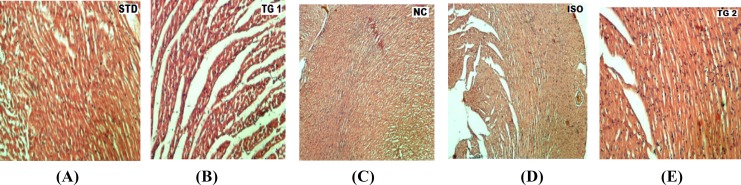
photomicrograph of heart section (10×, 10×10): a- Normal group (NC) showing normal cytoarchitecture; b- Isoprenaline (ISO) group shows focal myonecrosis with myophagocytosis and lymphocytic infiltration. In subendocardium vacuolar changes and prominent oedema along with chronic inflammatory cells are present; c- Standard (STD) group showing very lesser degree of myonecrosis, myophagocytosis and lymphocytic infiltration, oedema, and very little infiltration of inflammatory cells; d- Test group (TG1) showing decreased degree of myonecrosis and lesser infiltration of inflammatory cells but myophagophytosis and subendocardium vacuolar changes are present; e- Rat heart pretreated with test group 2 (TG2) (500 mg/kg) treated group showing little degree of myonecrosis and lesser infiltration of inflammatory cells as well as a decreased myophagophytosis and subendocardium vacuolar changes are present

## Discussion

Myocardial infarction remains a leading cause of morbidity and mortality worldwide. Prompt treatment of a heart attack is indispensable to prevent permanent damage and to save lives. In the traditional Indian medicinal system, a major role has been played by the aspect of cardioprotection.

 In this context, there is a need to reveal the cardioprotective activity of extract of *Bombyx mori* silk cocoon. ISO causes significant damage to myocardium and endocardium and a significant increase in the levels of serum marker enzymes such as AST, ALT, CK, LDH, and troponin-I (Suchalata et al., 2004 ▶). This might be due to the damage to the heart muscle, rendering the leakage of enzymes into the serum. The biochemical markers that are used widely in detection of myocardial necrosis are CK, LDH, and trasaminases. CK-MB has greater than 95% sensitivity and specificity for myocardial injury when measured between 24-36 hours. Estimation of elevated serum enzymes is a useful guide for necrosis of myocardium (Muralidharan et al., 2008 ▶). 

In the present study, pretreatment with alcoholic extract of *Bombyx mori* had significantly prevented ISO-induced myocardial damage, hypertrophy, and decreases the levels of the diagnostic marker enzymes as well as very effective in low and high doses, respectively, when compared with the ISO-treated rats against the standard drug, metoprolol (10 mg/kg). 

These results were also confirmed from the histopathological studies which showed normal architecture of the myocardium. There was no evidence of microscopic changes in the normal control group. The cardiac sections of the ISO– treated animals revealed degenerative changes in the muscle fiber and showed a coagulative necrosis. Pretreatment with cocoons extract and metoprolol exerted a protective effect as evident from the normal myofibrillar structures with striations.
